# Scale-Free Brain-Wave Music from Simultaneously EEG and fMRI Recordings

**DOI:** 10.1371/journal.pone.0049773

**Published:** 2012-11-14

**Authors:** Jing Lu, Dan Wu, Hua Yang, Cheng Luo, Chaoyi Li, Dezhong Yao

**Affiliations:** 1 Key Laboratory for NeuroInformation of Ministry of Education, School of Life Science and Technology, University of Electronic Science and Technology of China, Chengdu, China; 2 Sichuan Conservatory of Music, Chengdu, China; 3 Center for Life Sciences, Shanghai Institutes for Biological Sciences, Chinese Academy of Sciences, Shanghai, China; Beijing Normal University, Beijing, China

## Abstract

In the past years, a few methods have been developed to translate human EEG to music. In 2009, PloS One 4 e5915, we developed a method to generate scale-free brainwave music where the amplitude of EEG was translated to music pitch according to the power law followed by both of them, the period of an EEG waveform is translated directly to the duration of a note, and the logarithm of the average power change of EEG is translated to music intensity according to the Fechner's law. In this work, we proposed to adopt simultaneously-recorded fMRI signal to control the intensity of the EEG music, thus an EEG-fMRI music is generated by combining two different and simultaneous brain signals. And most importantly, this approach further realized power law for music intensity as fMRI signal follows it. Thus the EEG-fMRI music makes a step ahead in reflecting the physiological process of the scale-free brain.

## Introduction

Music and language define us as human [1]. Emotional expression and communication, through language or non-linguistic artistic expression, are recognized as being strongly linked to health and sense of well-being [2]. Therefore, as an artistic expression, music may represent human mind or mood.

In 1934, Adrian and Matthews attempted to listen to the brainwave signals via an amplified speaker [3]. During the past decades, scientists and artists found many methods to make an electroencephalogram(EEG) sonification, although it is difficult for composition to balance music principles and EEG features [4]. Meanwhile, in order to learn more about ourselves, researchers also used the deoxyribonucleic acid(DNA) [5], proteins [6], electromyograms (EMGs) [7] to compose music in the last century.

From the 1990s, various new EEG music generating rules were created [8]. One of them was to translate some parameters of EEG to the parameters of music [9], and another one was to utilize some characteristics such as the epileptic discharges to trigger specific music [10], or to link brain states to various music pieces through Brain Computer Interface [4].

In 2009, we proposed a method to translate EEG to music. The translation rules included the direct mapping from the period of an EEG waveform to the duration of a note, the logarithmic mapping of the average power (AP) change of EEG to music intensity according to the Fechner's law [11], and a scale-free based mapping from the amplitude of EEG to music pitch according to the power law [12]. However, in this method, the pitch and intensity were not independent enough under the translation rules as both pitch and intensity are related to EEG amplitude, so that the music was not strictly in accordance with the composition regulation in which pitch and intensity are usually not mutually related. Meanwhile, the intensity of music usually follows power law [13], however, the intensity of our previous brainwave music was obtained from the AP change of EEG within a time window, it didn't obey the power law (Shown in the following Figure 9). In this work, in order to imitate the general music composition better, we selected another brain information to represent the intensity instead of the EEG amplitude. As the intrinsic metabolic functional activities based functional magnetic resonance imaging(fMRI) is widely used to study the operational organization of the human brain [14], and fortunately, the fMRI blood oxygenation level dependent(BOLD) signal does follow the power law [15], thus the currently widely adopted fMRI may provide us a potential information for intensity of brainwave music. In fact, the fMRI BOLD signal is indirectly related to the electrical activities of a group of neurons by neuro-vascular coupling relation, thus it may reflect the brain mental state.

## Data and Methods

### 1 Ethics Statement

This study was approved by the Research Ethics Board at University of Electronic Science and Technology of China. All participants were asked to read and sign an informed consent form before participating in the study. After experiment, all participants received monetary compensation for their time and effort.

### 2 EEG-fMRI Data

For the simultaneous EEG-fMRI recordings, the subjects were a 31-year-old female (Subject A) and a 14-year-old female (Subject B). They were both in resting state and scanned in a 3T MRI scanner (EXCITE, GE Milwaukee, USA).

**Figure 1:**
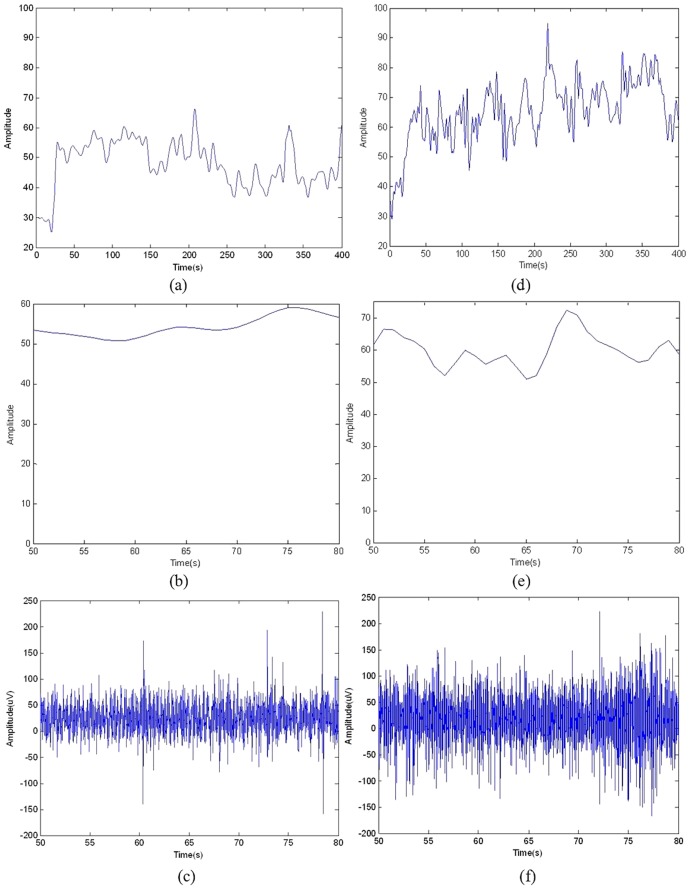
The EEG and fMRI signals. (a) and (d) the fMRI signals collected at MNI coordinate (15,−48, 60); (b) and (e) pieces of signals in (a) and (d) for the following music compositions; (c) and (f) 30 s simultaneous EEGs collected at scalp Cz, respectively for Subjects A and B.

For composing music, the EEG recordings were re-referenced to zero with a software called REST developed in our laboratory [16], [17]. In this work, we chose the EEG at Cz electrode for brainwave music composition, which is at the central of the head and is a channel less affected by the body movement, and took the fMRI signal at the MNI(Montreal Neurological Institute) [18] coordinate (15,−48, 60), which was just below the electrode position Cz. In this way, we assumed the EEG and fMRI signals were almost from the same neural mass. The signals used in this work are illustrated in Figure 1.

### 3 EEG-fMRI brain Music

Music note consists of four characters, pitch, timbre, duration and intensity. In this work, we paid special attention to pitch and intensity. And timbre was fixed with piano, which could be changed according to person's hobbies,while the duration was determined by the period of an EEG waveform.

### 3.1 Pitch

In this study, we still adopt the power law rule between the amplitude (*Amp*) of an EEG waveform and the *Pitch* of a musical note [12],
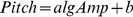
(1)In equation (1), *b* is the maximum value of all pitches. Parameter *a* denotes the scale characteristics, and it is determined by the following detrended fluctuation analysis (DFA) [19], [20].

For a discrete time series 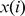, the first step of DFA is to subtract the mean from the series and then create a new series by integration:
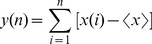
(2)where 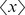
denotes the mean of the time series. Next, series
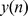
is divided into a number of segments with length k (k represents the time scale of observation). For each of these segments, a local least-squares linear fit is conducted, and the resulted piece wise linear fit function is designated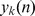. Then the root mean square fluctuation, with different scale variable k of 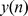 after detrended by 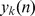 is calculated by:
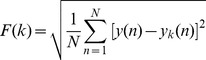
(3)In the final step, the logarithm of 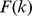
is plotted as a function of the logarithm of the time scale *k*. If the time series 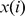
is self-similar, the scale-free property of a fractal geometry, this plot will display a linear scaling region, and the slope of the plot, *alpha* = 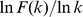, is called the scaling or self-similarity coefficient. If *alpha* = 0.5, 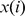
is uncorrelated white noise, if *alpha* = 1.5, 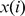 is Brownian noise, and if *alpha* = 1, 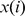
is a 1/*f* power-law process widely existed in the real world [21].

**Figure 2:**
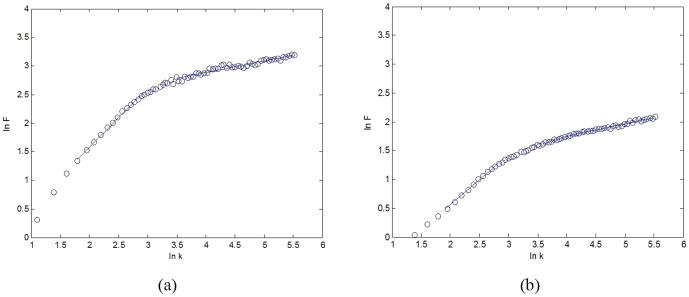
EEG detrended fluctuation analysis and Scale-free regions. (a) Subject A, the left region alpha = 1.022, and the right region alpha = 0.195; (b) Subject B, the left region alpha = 0.878, and the right region alpha = 0.215. Here k is the window size, and F(k) is the fluctuation from the local trends in windows.

With the obtained *alpha* from DFA, we defined the parameter *a* in equation (1) as *a = -c/alpha*, where *c* is a constant. In order to ensure the pitch vary from 0 to 96 in the range of the 128 pitch steps in MIDI (Musical Instrument Digital Interface), we chose *c* = 40 [12]. For the data of Subjects A and B, there are twoscale-free regions for each subject (Figure 2) [12], [21], [22].

### 3.2 Intensity

#### 1) Defined with EEG AP

In our previous work [12], the intensity of a music note(*MI*) was proportional to the logarithm of the *AP* change according to the Fechner's law [11].
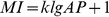
(5)Where *l* and *k* are two constants. In this approach, *MI* was partly related with *Pitch,* since both of them are defined with something related to the amplitude of EEG.

#### 2) Defined with fMRI signal

**Figure 3:**
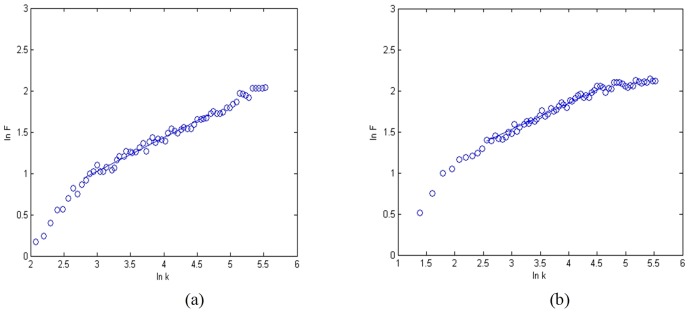
fMRI signal detrended fluctuation analysis and Scale-free regions. (a) Subject A, alpha = 1.013; (b) Subject B, alpha = 0.875. Here k is the window size, and F(k) is the fluctuation from the local trends in windows.

**Figure 4:**
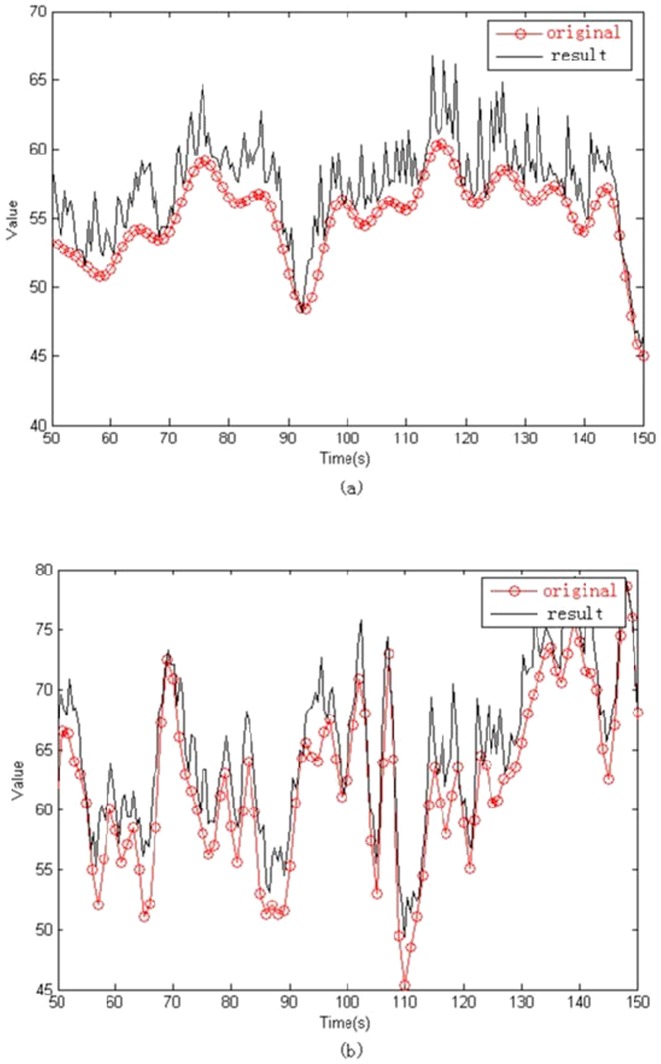
Fractal Interpolation of the fMRI signals. Points indicate the original sample, the solid lines indicate the interpolated function.(a) and (b) for Subject A and B, respectively.

In this work, we proposed to adopt fMRI signal instead of the AP change of EEG to represent intensity of music. And as fMRI signal follows power law (Figure 3) [15], the resulted intensity of EEG-fMRI music would follow the power law too. Furthmore, as the sample rate of fMRI (about 2 seconds) is much lower than the usual music tempo, an interpolation step is necessary. In order to keep the power law and the underneath fractal structure, we adopted a fractal interpolation algorithm [23] to increase the sample rate. Figure 4 illustrates the interpolation results of the data used in this work. The new sample rate of the interpolated series is 1 second, which is close to the rate of a peaceful music.

### 3.3 The mapping rules

**Figure 5:**
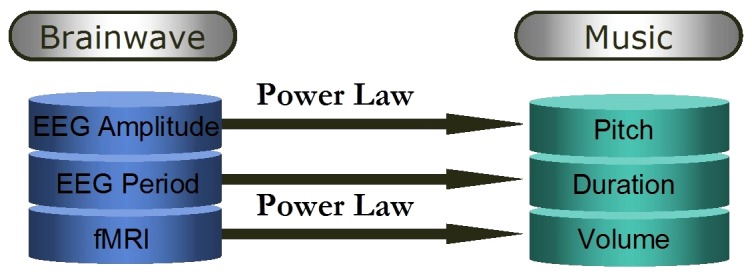
Composition rules of EEG-fMRI music. The amplitude, waveform period, and fMRI signal are mapped to pitch, duration and intensity, respectively. The mappings from amplitude to pitch and from fMRI signal to intensity are based the power law.

The mapping rules between the brain physiological signals and the attributes of a music note are shown in Figure 5, where the fMRI signal reflected the BOLD signal, the EEG reflected the neural electrical activities. To denote the difference, we take the new music as EEG-fMRI music, whose intensity is fMRI based, and the previous one as EEG music, its intensity is EEG AP change based [12].

## Results

### 1 EEG music

**Figure 6:**
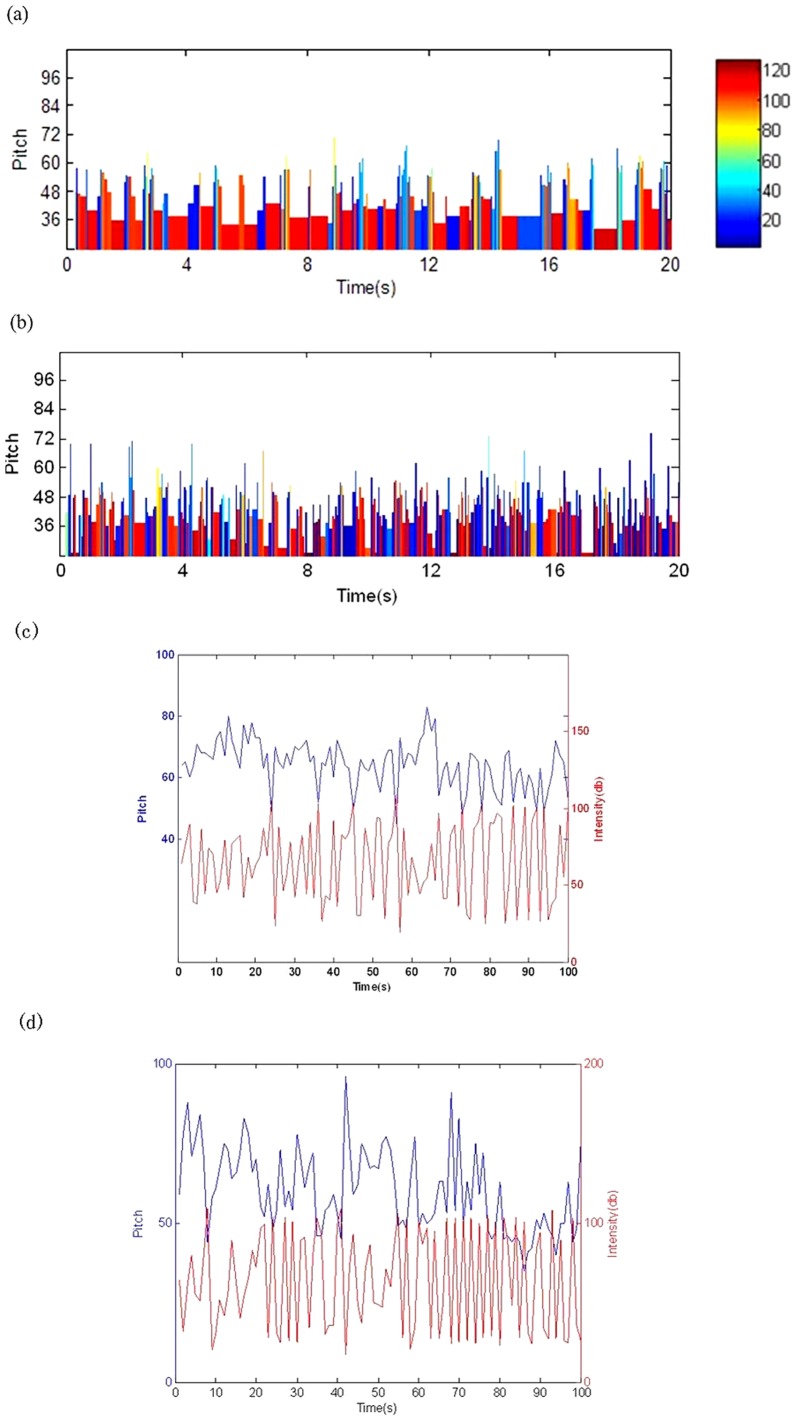
EEG music. Each column denotes a note, where the pitch (height of a column) is determined by Equation (1), the duration (width of a column) is defined as the period of an EEG wave, and the intensity (color of a column) is determined by Equation (5). (a) EEG music of Subject A with *b*_1_ = 96, *b*_2_ = 108,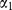 = 1.02,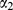 = 0.20; (b) EEG music of Subject B with *b*_1_ = 96, *b*_2_ = 108,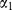 = 0.88,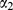 = 0.21; (c) the relation between the pitch and intensity of the EEG music of Subject A with correlation coefficient = 0.427 (p<0.05); (d) the relation between the pitch and intensity of the EEG music of Subject B with correlation coefficient = 0.494(p<0.05).

With the rules defined in paper [12], the EEG music (Figure 6(a)–(b)) was obtained from the EEG data (Figure 1). The EEG music (Audio S1, S2) sounds reasonably. However, as the pitch and intensity are derived from the amplitude and the induced AP, they were correlated significantly (Figure 6(c)–(d)).

### 2 EEG-fMRI music

**Figure 7:**
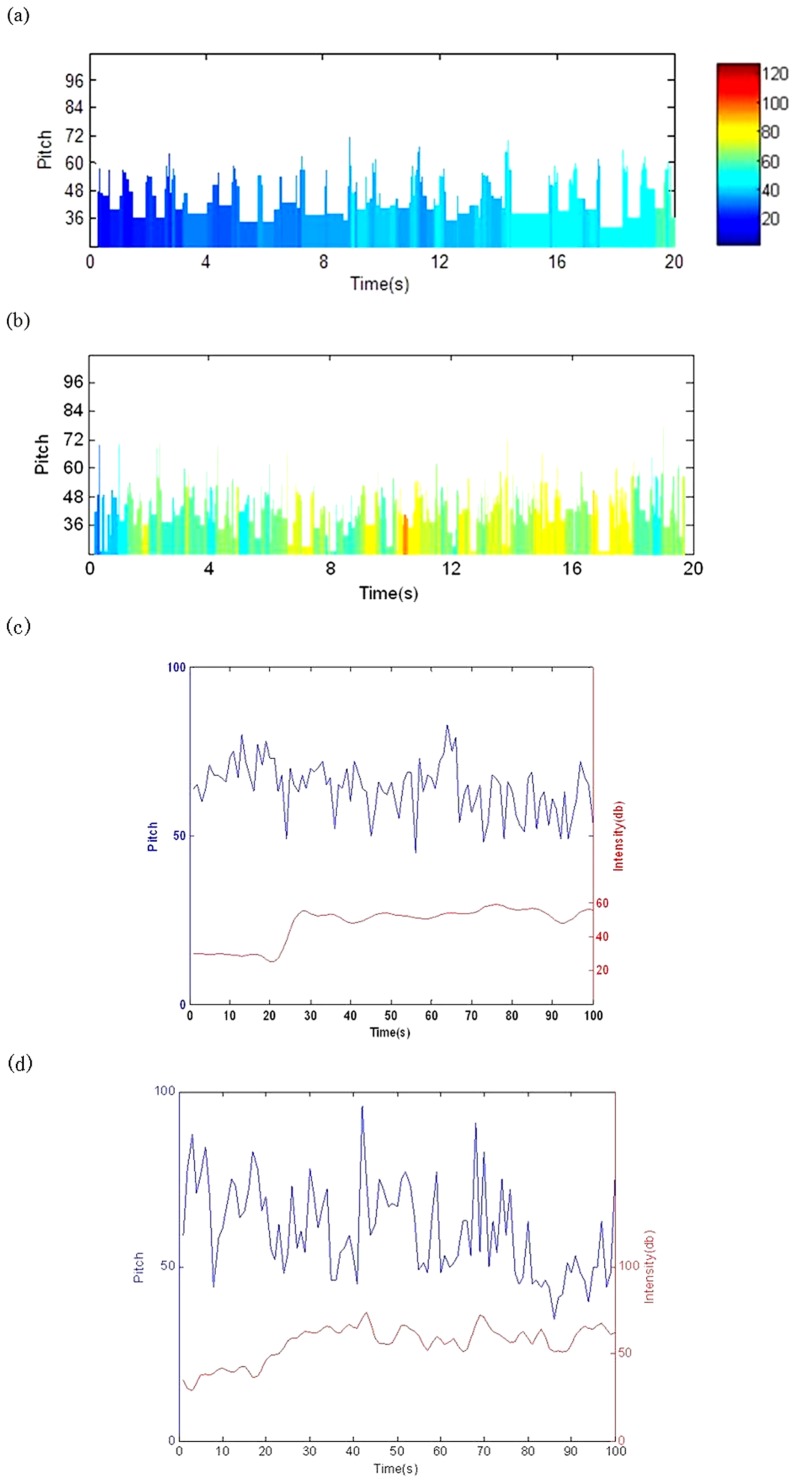
EEG-fMRI music. Each column denotes a note, where the pitch (height of a column) is determined by Equation (1), the duration (width of a column) is defined as the period of the EEG wave, and the intensity (color of a column) is determined by fMRI signal. (a) EEG-fMRI music of Subject A; (b) EEG-fMRI music of Subject B. The parameters adopted here are the same with Figure 6; (c) The relation between the pitch and intensity of the EEG-fMRI music of Subject A with correlation coefficient = 0.0048 (p>0.05); (d) The relation between the pitch and intensity of the EEG-fMRI music of Subject B with correlation coefficient = 0.0095(p>0.05).

**Figure 8:**
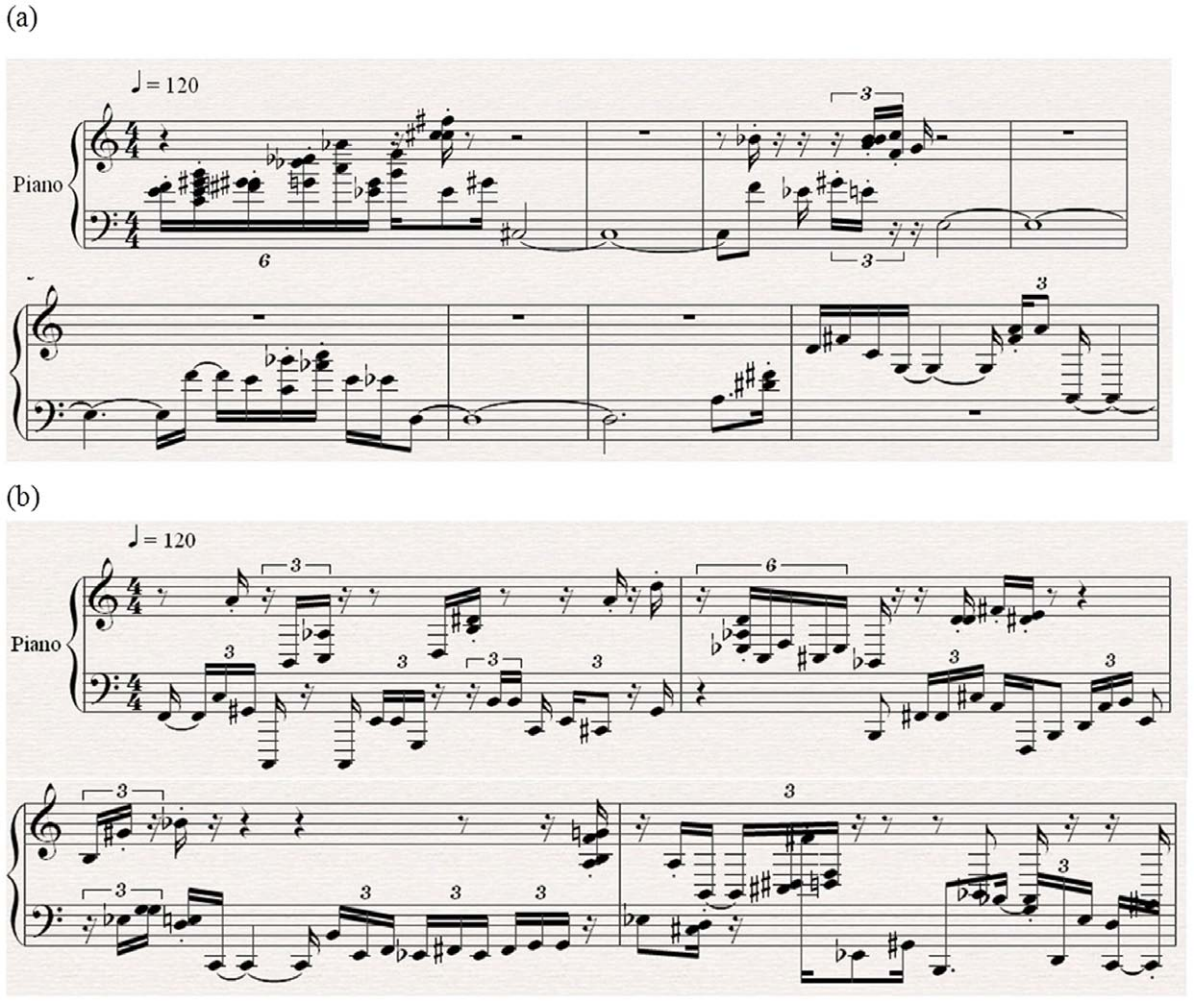
Illustration of the score of EEG-fMRI music (Printed by Sibelius 4.0). (a) Score of brain music of Subject A, (b) score of brain music of Subject B.

With the newly defined translation rules (Figure 5), the EEG-fMRI music (Figure 7, Audio S3, S4)was obtained from the EEG-fMRI data(Figure 1). In these music, as the pitch and intensity were defined separately by EEG amplitude and fMRI signal, they were not correlated directly. In fact, the correlation coefficient between pitch and intensity of the EEG-fMRI music is smaller than 0.01 (p>0.05) (Figure 7), which is much smaller than the case in EEG music (Figure 6), and this phenomenon is similar to a general man-made music. Figure 8 shows two pieces of score of EEG-fMRI music of the two subjects, and as music score only records the pitch and duration, the scores of EEG-fMRI music are the same with the EEG music, and the difference between them can only be recognized by listening to the Audios (See Audio S1, S2, S3, S4).

### 3 Difference between EEG and EEG-fMRI music

In order to evaluate the difference between EEG music and EEG-fMRI music, 10 persons who have received music training for at least 3 years were invited to listen to the two subjects' music. In the test, 5 of them listened to the EEG music first and the other 5 listened to EEG-fMRI music first. For the question that which intensity change was quicker, 8(9) of them chose EEG music of Subject A(B), and for the question that which intensity change was slower, 9(9) of them chose EEG-fMRI music of subject A(B) (Table 1). The average identification of EEG music is 85%, and the average identification of EEG-fMRI music is 90%. Based on t-test, the overall evaluation is significant (EEG Music, T = 2.30, P<0.05; EEG-fMRI Music, T = 4.017, P<0.05).

**Table 1:** Intensity Variation recognition by 10 volunteers.

Subject A (B)	Quick	Slow	Similar	Accuracy
EEG music	8(9)	1(0)	1(1)	80%(90%)
EEG+fMRI music	1(0)	9(9)	0(1)	90%(90%)

These results indicate that the two kinds of music are quite different in intensity movement. And they all reported that the intensity change speed of the EEG-fMRI music was more close to usual human made scores.

### 4 Power law of the EEG and EEG-fMRI music

#### 4.1 Power law of the EEG music

**Figure 9:**
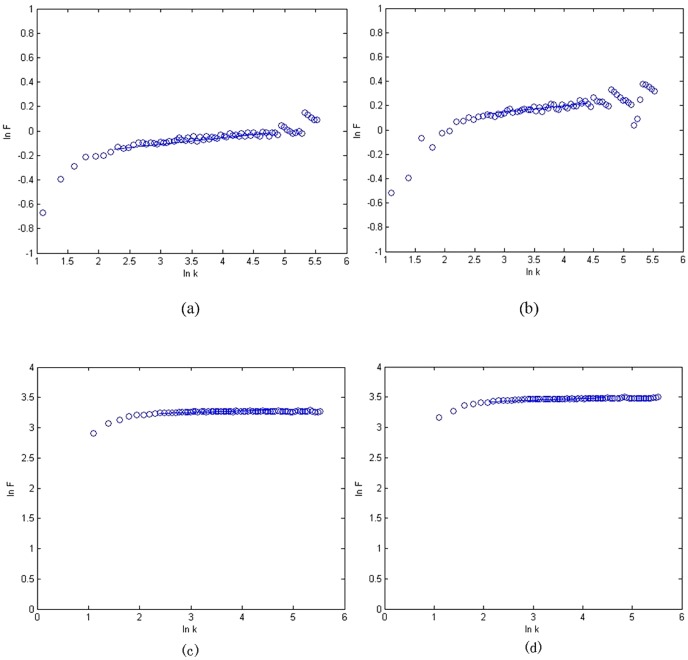
Detrended fluctuation analysis of EEG-music duration and volume. (a)–(b) DFA of EEG music note duration for subjects A and B, respectively. For Subject A, alpha = 0.109, for Subject B, alpha = 0.056. (c)–(d) DFA of EEG music note volume for subjects A and B, respectively. For Subject A, alpha = 0.123, for Subject B, alpha = 0.082. Here k is the window size, and F(k) is the fluctuation from the local trends in windows.

Based on the translation rule (Equation.(1)) and the scale-free property of the EEG amplitude data (Figure 2), the pitch of EEG music obeys the power law rule [12].For the intensity and duration of EEG music, Figure 9 shows their DFA results, respectively, they clearly indicate that the scale index of each case *(alpha)* of each case is much smaller than 0.5, thus they all belong to “uncorrelated white noise”. This fact means that for EEG music only pitch has imitated the usual man-made music.

#### 4.2 Power law of the EEG-fMRI music

According to Figure 3, the fMRI signal, which represents the intensity of the EEG-fMRI music, obeys the power law rule. Therefore, from EEG music to EEG-fMRI music, not just the pitch but also the volume of music obeys the power law rule.

## Discussions and Conclusions

### 1 Intensity and pitch

For EEG music [12], due to the quick change of the EEG state, the intensity of EEG music changed quickly and abruptly (Figure 6, Audio S1, S2), and this is not the usual case in man-made music. To reduce the gap, in this work, we chose another brain information, the fMRI signal, serving as the intensity information source. As the EEG-fMRI intensity evolution is smooth and leisure,the resulted EEG-fMRI sounds more close to the man-made real music (Figure 7, Audio S3, S4).

Besides, pitch and intensity, as two important factors in music composing, should be independent to each other in general. However, in the above EEG music, the amplitude of EEG is translated to pitch, and the AP change of EEG was used to represent the intensity of music, thereafter they two are not properly separated with each other. While in the above new EEG-fMRI music, the fMRI BOLD signal was used to represent intensity, it is almost independent to the EEG amplitude, untied the unreal close relation between pitch and intensity in EEG music thus better fit the usual composing regulation.

About the future of the EEG-fMRI music, the fMRI signal needs to be further carefully selected. As different frequency band of the fMRI signal may have different functional role, other than the adopted 0.01–0.08 Hz fMRI can be evaluated

### 2 Scale free music of the brain

Either EEG or fMRI is a physiologic signal. If we translate them to music directly, the physiological information sounds completely insert into the ‘music’, however, sucha ‘music’ may be just like noise. On the other hand, if we just use some EEG feature to trigger a man-made music piece, the music might be very pleasurable, but the physiological information was less involved. Therefore, a valuable and reasonable method would be a trade-off of these two extremes. In our approaches, both the previous EEG music and this new EEG-fMRI music, we assume the translation should follow some common rules obeyed by both brain signal and music. We believe that this is the correct way to understand human body through auditory. In our work, we pay special attention on scale-free phenomena, and as the scale-free phenomena exist widely in nature, including music and neural activities, it would be a reflection of the underneath truth of the mental state of the brain. The EEG-fMRI makes a step ahead the previous EEG music by extending the pitch scale-free of EEG music to both pitch and intensity scale-free of EEG-fMRI music, thus it provides us a new window to look inside the brain.

### 3 Spatio-temporal music

FMRI, which reflects the brain inherent metabolic activities, has a high spatial resolution,from which the music generated may display more details in spatial than any other currently available physiologic signal. Nevertheless, the EEG, collected on the surface of brain, is of high temporal resolution, could reflect brain's instantaneous activities. Therefore, it would be an interesting topic to develop a fusion method which combine EEG and fMRI to get the specific EEG-fMRI activities inside the brain [24], [25]. Then it would be more reasonable to make a vivo spatio-temporal music with EEG and fMRI which was derived from the same location inside the brain in the future.

### 4 Physiological music and man-made music

**Figure 10:**
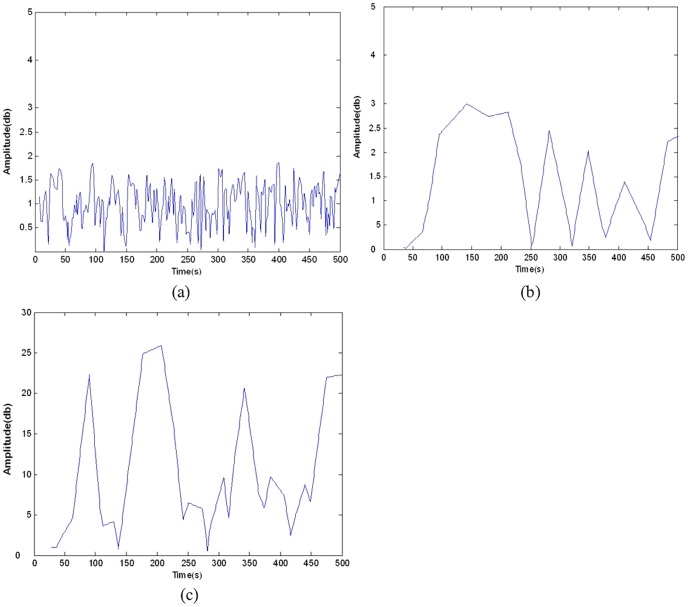
The envelope variation ranges of music waveform. (a) A piece of real music ‘Nocturnes’, composed by Mozart, (b) EEG-fMRI music and (c) EEG music.

Table 1 reveals the distinct difference between the EEG and EEG-fMRI music, and the all subjects also reported that the intensity change speed of the EEG-fMRI music was more close to the usual human made scores. Here in order to display the intensity characteristics, we recorded the two types of music of Subject A by SONAR 6.0 and measured the variations of the envelope of the music waveforms, which represented the intensity changes of music. As contrast, we also measured a piece of real music ‘Nocturnes’, composed by Mozart. The results are shown in Figure 10. It is clear that the variation ranges of the intensity of the EEG-fMRI music waveform's envelope are similar with that of Mozart's music. Both of them are below 5 db. However, the changes of the intensity of EEG music are much wider. This fact means that the EEG-fMRI music translation method is better in mimic the real music. In addition, we could argue that the EEG-fMRI music do may better reflect the physiological brain process as the experiment state of Subject A was on resting state, and the intensity of her EEG-fMRI music does change slightly, just like a really peaceful music ‘Nocturnes’.

### 5 Conclusion

In this work, we proposed a new method to translate both brain EEG and fMRI signals to music to better reflect the internal functional activities of the brain under the power law framework. The resulted music sounds better in mimic the man-made music intensity change. The brain music, as one of the human brain's intelligence product, embodies the secret of brain in an artistic style, provides the platform for scientist and artist to work together to understand ourselves, and it is also a new interactive link between the human brain and music. We hope the on-going progresses of the brain signals based music will properly unravel part of the truth in the brain, and then to be used for clinical diagnosis and bio-feedback therapy in the future.

## Supporting Information

Audio S1 30 s EEG music of Subject A from the resting state.
(MP3)

Audio S2 30 s EEG music of Subject B from the resting state.
(MP3)

Audio S3 30 s EEG-fMRI music of Subject A from the resting state.
(MP3)

Audio S4 30 s EEG-fMRI music of Subject B from the resting state.
(MP3)
